# The synergistic pathogenic mechanism of Brucella melitensis M5-90 effector protein BspC in regulating host cell apoptosis, inflammation and oxidative stress

**DOI:** 10.1099/jmm.0.002092

**Published:** 2025-12-05

**Authors:** Shuanghong Yin, Li Li, Lan Zhu, Jinke He, Yang Liu, Longwei Xi, Hong Mao, Xiaoyu Deng, Jihai Yi, Junbo Zhang

**Affiliations:** 1 College of One Health, Tongren University, Tongren, Guizhou, PR China; 2 College of Agroforestry Engineering and Planning, Tongren University, Tongren, Guizhou, PR China; 3 Department of Basic Medicine, Xinjiang Second Medical College, Kelamayi, Xinjiang, PR China; 4 College of Basic Medicine, Hunan University of Medicine, Huaihua, Hunan, PR China; 5 College of Animal Science and Technology, Shihezi University, Shihezi, Xinjiang, PR China; 6 Guizhou Provincial Key Laboratory for Biodiversity Conservation and Utilization in the Fanjing Mountain Region, Tongren University, Tongren, Guizhou, PR China

**Keywords:** *Brucella*, effector protein BspC, interacting protein, transcriptome sequencing, yeast two-hybrid

## Abstract

**Introduction.** Brucellosis is a zoonotic contact infectious disease caused by *Brucella*. Recently, researchers screened a series of *Brucella* effector proteins and identified the functions of most of them, which play important roles in the intracellular survival of *Brucella*.

**Hypothesis.** We hypothesized that the BspC protein from *Brucella melitensis* M5-90 plays a significant role in the intracellular proliferation of *Brucella*.

**Aim.** This study aimed to investigate the role of the *Brucella melitensis* M5-90 effector protein BspC in host cell processes, specifically its interaction with host proteins, and its effects on apoptosis, inflammation, oxidative stress, and global gene expression.

**Methodology.** First, host proteins that interacted with BspC were identified from the cDNA library of a mouse macrophage cell line (RAW264.7) by yeast two-hybrid technology, and the key interacting proteins were confirmed by cotransformation. The pTT5-BspC recombinant plasmid was subsequently constructed and transfected into HEK293T cells. The expression of apoptosis-related proteins was assessed by Western blotting, the apoptosis rate was analysed by flow cytometry, the subcellular localization of proteins was observed by laser confocal microscopy and cytokines and oxidative stress indicators were assessed by ELISA and other kits. Furthermore, RNA-seq transcriptome sequencing was used to analyse the effects of BspC on the host gene expression profile.

**Results.** Four host proteins (FDX1, DNAJA1, HSPA5 and PTPN2) that interacted with BspC were identified from the RAW264.7 cell cDNA library by yeast two-hybrid technology, and their interactions were confirmed by cotransformation. BspC protein expression in HEK293T cells significantly promoted cell apoptosis, as indicated by the upregulation of proapoptotic proteins (Bax, p53 and Caspase-3) and the downregulation of the antiapoptotic protein Bcl-2. Immunofluorescence staining revealed that BspC was localized in the cytoplasm and nucleus. In addition, BspC induced the secretion of proinflammatory cytokines (TNF-α, IL-1β and IL-6) and lactate dehydrogenase and reduced malondialdehyde levels by increasing the activities of SOD, CAT, GSH-PX and GSH, suggesting its regulation of the oxidative stress response. Transcriptome analysis revealed that BspC expression induced differential expression in 796 genes (209 upregulated and 587 downregulated), which were significantly enriched in the ECM-receptor interaction and MAPK and NF-κB signalling pathways.

**Conclusion.** BspC may promote the immune escape and pathogenicity of *Brucella* by interfering with host cell apoptosis, the inflammatory response and the redox balance.

## Introduction

Brucellosis is a zoonotic contact infectious disease caused by *Brucella*, which can cause abortion, orchitis, arthritis and other symptoms in animals [1]; in humans, it can lead to undulant fever, excessive sweating, muscle pain and general discomfort. *Brucella* is classified into six classic species on the basis of the different hosts they infect: sheep, swine, bovine, canine, Sardinian vole and goat [2]. *Brucella* infection shares the same pathological and physiological characteristics in both animal and human cells, and the survival, proliferation and parasitism of *Brucella* occur through intracellular circulation [3].

The type IV secretion system of *Brucella* is recognized as an important virulence regulatory factor that is closely related to its intracellular survival [4,5]. *Brucella* releases effector proteins into host cells through the type IV secretion system, which can induce the apoptosis of host cells, trigger inflammation and play a significant role in innate and adaptive immunity [6]. Studies have shown that after *Brucella* invades cells, it first forms a *Brucella*-containing vacuole (BCV) to evade lysosomal degradation. During the entire intracellular transport process of BCV, the type IV secretion system can secrete multiple effector proteins, playing crucial roles in regulating the maturation and intracellular transport of BCV, evading host immune surveillance and maintaining persistent infection [7].

Recently, researchers screened a series of *Brucella* effector proteins and identified the functions of most of them, which play important roles in the intracellular survival of *Brucella* [8,9]. VceC was found to inhibit apoptosis via the CHOP pathway induced by endoplasmic reticulum stress, providing protection to enable *Brucella* to persistently infect host cells [10]; studies have shown that a strain of *Brucella abortus* with deletion of the VceA gene can inhibit apoptosis in human embryonic trophoblast cells [11]. The strain with deletion of the BspJ gene can induce apoptosis in macrophages and inhibit the proliferation of *Brucella* within host cells [12]. The deletion of the BspG gene reduces the intracellular proliferation ability of *Brucella* [13]. However, the mechanism and function of the effector protein BspC remain unclear.

In this study, we used yeast two-hybrid technology to screen a cDNA library of RAW264.7 cells and identify host target proteins that interacted with the *Brucella* effector protein BspC. We utilized molecular biology and cell biology techniques to systematically study the effects of BspC on host cell apoptosis (related protein expression and the percentage of apoptotic cells), inflammatory factor secretion and oxidative stress indicators. We used transcriptome sequencing to analyse the differentially expressed genes (DEGs) regulated by BspC and their related signalling pathways. We aimed to clarify the mechanism of BspC in the *Brucella* infection process at three levels, namely, molecular interaction, cell function and gene regulation, to provide an important theoretical basis for an in-depth understanding of the pathogenic mechanism of *Brucella*.

## Methods

### Main materials

The yeast two-hybrid system components, including Y2H Gold competent cells, plasmids (pGBKT7, pGADT7, pGBKT7-53 and pGADT7-T), a yeast plasmid miniprep kit and nutritionally deficient media, were from Clontech (USA). Molecular biology reagents (restriction enzymes, T4 DNA ligase, gel extraction kit, DNA markers and protein loading buffer) were purchased from TaKaRa (China). Cell culture reagents (DMEM, FBS and trypsin) were obtained from GIBCO (USA). The PEI transfection reagent was from Sigma (USA), and the ECL kit was from Thermo Scientific (USA). PVDF membranes were purchased from Millipore (USA), and DAPI was from Solarbio (China). All antibodies were from Proteintech (USA). Cytokine and lactate dehydrogenase (LDH) assay kits were from Jiangsu Jingmei Biotechnology (China), and oxidative stress parameter kits [superoxide dismutase (SOD), catalase (CAT), malondialdehyde (MDA), GSH-PX and glutathione (GSH)] were from Nanjing Jiancheng Bioengineering Institute (China).

### Construction and assessment of the bait plasmid and toxicity and autoactivation assessments of the BspC bait protein

The DNA sequence encoding the BspC gene (BM590_RS03990) from *B. melitensis* M5-90 (accession no: NC_017246) was codon-optimized and synthesized by GenScript Biotech Corporation with EcoRI and BamHI restriction sites incorporated at the 5′ and 3′ ends. The synthesized fragment was then digested with EcoRI and BamHI (TaKaRa) and ligated into the similarly digested pGBKT7 vector using T4 DNA ligase (TaKaRa) to generate the pGBKT7-BspC bait plasmid. After construction, double enzyme digestion was performed with EcoRI and EcoRV, and the products were sent for sequencing verification. The correctly identified bait plasmids pGBKT7-BspC and pGADT7 were cotransformed into Y2H Gold competent yeast cells; the recombinant plasmids pGBKT7-53 and pGADT7-T were cotransformed into Y2H Gold competent yeast cells as positive controls, and the pGBKT7-Lam and pGADT7-T plasmids were cotransformed into Y2H Gold competent yeast cells as negative controls. The transformed cell suspensions were spread on SD/-Leu/-Trp/X-α-gal (DDO/X), SD/-Leu/-Trp/-His/X-α-gal (TDO/X) and SD/-Leu/-Trp/-His/-Ade/X-α-gal/AbA (QDO/X/A) solid culture plates and incubated at 30 °C for 3–5 days to observe the growth status of colonies and assess the toxicity and autoactivation of the BspC bait protein expressed by the yeast cells.

### Yeast two-hybrid screening of host proteins that interact with the BspC protein

A yeast two-hybrid assay was used to screen proteins that interact with BspC in the cDNA library of RAW264.7 cells. The pGBKT7-BspC plasmid (5 µg) and the cDNA library of RAW264.7 cells (10 µg) were cotransformed into Y2H Gold competent yeast cells; the cotransformation of pGBKT7-53 and pGADT7-T into Y2H Gold competent yeast cells was used as a positive control, and the cotransformation of pGBKT7-Lam and pGADT7-T into Y2H Gold competent yeast cells was used as a negative control. The transformed yeast mixture was spread on TDO/X screening plates and incubated at 30 °C for 3–5 days. Single colonies were counted to complete the primary screening. The blue colonies on the primary screening plates were transferred to QDO/X/A secondary screening plates for further screening. The QDO/X/A plates were incubated at 30 °C for 3–5 days. The blue colonies were picked and cultured in QDO liquid medium at 30 °C for 24 h. The plasmids were extracted and identified by PCR. The positive plasmids were sequenced by Qiangke Biotechnology Co., Ltd. The sequencing results were analysed by blast in the NCBI database.

### Cross-verification test of host proteins that interacted with the BspC protein

The five positive plasmids were cotransformed with pGBKT7-BspC (100 ng) into Y2H Gold competent yeast cells. A positive control (pGBKT7-53+pGADT7-T), a negative control (pGBKT7-Lam+pGADT7-T) and a blank plasmid control (pGBKT7-BspC+pGADT7) were used. The transformed yeast mixture was spread on DDO (SD/-Leu/-Trp) and QDO/X/A plates and incubated at 30 °C for 3–5 days. The growth of the yeast was observed. The blue single colonies were resuspended in 0.9% NaCl solution and 10-fold serially diluted (10^1^–10^4^). Then, 10 µl of each dilution was spread on DDO and QDO/X/A plates and incubated at 30 °C for 3–5 days. The growth of the yeast in each group was observed.

### Construction and identification of the recombinant expression plasmid pTT5-BspC

The BspC gene sequence of *Brucella* was introduced into the restriction sites (EcoRI/HindIII) and synthesized by General Biosystems (Anhui) Co., Ltd. (China). It was subsequently cloned and inserted into the eukaryotic expression vector pTT5 and transformed into DH5α competent cells. Positive clones were picked and cultured overnight. The plasmids were extracted and verified by EcoRI/HindIII digestion. The plasmids were assessed by 1.0% agarose gel electrophoresis. The resulting plasmids were subsequently sent to BGI Tech Solutions Co., Ltd., for sequencing. The correct recombinant plasmid was designated pTT5-BspC.

### Transfection of the recombinant expression plasmid pTT5-BspC and assessment of protein expression

One day before transfection, HEK293T cells were seeded in 10 cm culture dishes and cultured in DMEM containing 10% FBS until the cell density reached ~70% at the time of transfection. For transfection, 60 µl of PEI transfection reagent and 20 µg of plasmid were diluted in 800 µl of medium, mixed and incubated at room temperature for 20 min. The volume was subsequently adjusted to 3 ml with medium. The original medium was aspirated, and the transfection mixture was added. The cells were cultured at 37 °C for 5–6 h. Then, 2 ml of complete medium was added, and the cells were cultured for another 48 h. The transfected cells were collected, and proteins were extracted. Protein expression was analysed by Western blotting with rabbit anti-HA and anti-GAPDH primary antibodies, and an HRP-conjugated AffiniPure goat anti-rabbit IgG (H+L) secondary antibody was used for detection.

### Western blotting

A total of 1×10⁷ transfected cells were collected and mixed with RIPA buffer (50 mM Tris-HCl, pH 8.0, 150 mM sodium chloride, 1 % NP40, 0.5% sodium deoxycholate, 0.1% SDS, 1 mM EDTA, protease inhibitors). Cell lysates were obtained by gentle pipetting and incubation on ice for 60 min. The cells were lysed on ice for 20 min and then sonicated for 2 min. The lysate was centrifuged at 4 °C and 12,000 r min^−1^ for 10 min. The supernatant was collected to obtain the total cellular protein. The protein samples were separated by 10% SDS‒PAGE and transferred to PVDF membranes at 100 V for 1 h. The membranes were blocked with 5% skim milk at room temperature for 2 h and subsequently incubated overnight at 4 °C with the following primary antibodies: rabbit anti-Bax (Cell Signaling Technology, #2772, USA, 1 : 1000), anti-Bcl-2 (Cell Signaling Technology, #28150, USA, 1 : 1000), anti-p53 (Wuhan Sanying Biotechnology, 80077-1-RR, China, 1 : 5000), anti-Caspase-3 (Abcam, ab184787, UK, 1 : 1000) or anti-GAPDH (Abcam, ab181602, UK, 1 : 1000). After washing, the membranes were incubated with HRP-conjugated goat anti-rabbit secondary antibody (ASPEN, AS1107, USA, 1 : 10000) for 1.5 h at room temperature. The ECL developing solution (A solution:B solution=1 : 1) was mixed and applied to evenly cover the membrane surface, followed by development in a dark room. The grey values of the protein bands were analysed by ImageJ, and the relative expression was expressed as the ratio of the grey values of the target protein to GAPDH. Statistical analysis was performed.

### Assessment of the effect of the BspC protein on cell apoptosis

Cells transfected with pTT5-BspC for 24 and 48 h were collected and washed with PBS, and 100 µl of cells was added to each tube. Then, 5 µl of annexin V-FITC was added to the tube and incubated at room temperature in the dark for 10 min. After that, 5 µl of PI was added, and the mixture was incubated for 5 min. PBS was added to a total volume of 500 µl, followed by gentle mixing. The apoptosis rate was assessed by flow cytometry.

### Detection of the subcellular localization of the BspC protein

The pTT5-BspC plasmid was transiently transfected into 293T cells. After 24 h of transfection, ~20% of the transfected cells were seeded into glass-bottom culture dishes. After the cells had adhered to the dish, the culture medium was discarded, and the cells were washed twice with PBS. Then, the cells were fixed with 4% paraformaldehyde for 20 min and treated with 0.2% Triton X-100 for 10 min. Next, the cells were blocked with 2% BSA for 1 h, and rabbit anti-HA primary antibody (diluted 1 : 100) was added and incubated at 4 °C overnight. The next day, CY3-labelled goat anti-rabbit secondary antibody (diluted 1 : 100) was added and incubated at 4 °C for 2 h. Finally, DAPI was added to stain the nuclei, and the mixture was incubated at room temperature in the dark for 20 min. The cells were mounted with anti-fluorescence quenching mounting medium and observed and photographed by laser confocal microscopy.

### Assessment of the effect of the BspC protein on cytokine secretion

Cells transfected with pTT5-BspC were cultured at 37 °C in a 5% CO_2_ incubator for 24 and 48 h. The supernatants of the cell culture media were collected, and the levels of TNF-α, IL-1β, IL-6 and LDH were determined by ELISA. The experiment was repeated three times, and the empty plasmid pTT5 transfection group was used as the control. The standard curve was drawn according to the kit instructions.

### Assessment of the effects of the BspC protein on cell biochemical indicators

Cells transfected with pTT5-BspC were cultured at 37 °C in a 5% CO_2_ incubator for 24 and 48 h. Then, 400 µl of precooled physiological saline was added to the cell pellet, and the cells were treated with a high-speed grinder. The mixture was centrifuged at 2,500 r min^−1^ for 10 min, and the SOD, CAT, MDA, GSH-PX and GSH contents in the supernatant were determined with kits.

### RNA extraction, library preparation and sequencing

Cells in the pTT5-BspC experimental group (C1) and the pTT5 control group (B1) were seeded into six-well plates at a density of 5×10^5^ cells per well and cultured in an incubator for 48 h. Then, 1 ml of TRIzol was added to each well and mixed several times until the mixture was not viscous. The RNA was extracted from the mixture. The purity, concentration and integrity of the extracted RNA were assessed. The integrity of the RNA was assessed by agarose gel electrophoresis, and the purity and concentration of the RNA were assessed by a microultraviolet spectrophotometer.

### Validation of DEGs

The cells from the pTT5-BspC transfection group (three groups), the pTT5 group (two groups) and the normal cell group (one group) were cultured at 37 °C in a 5% CO_2_ incubator for 24 h. Total RNA was extracted, and cDNA was synthesized. Five DEGs were randomly selected, and PCR primers were designed with Primer Premier 5.0 software and synthesized by General Biosystems (Anhui) Co., Ltd. (China) (Table 1). GAPDH was used as the internal reference gene to verify the target genes by quantitative reverse transcription PCR (qRT-PCR). Each sample was set up in triplicate, and the relative expression levels of the genes were calculated by the 2^-△△CT^ method. The results are expressed as the means±sd.

**Table 1.** Information of qRT-PCR primers

Genes	Accession no.	Primer sequences (5′−3′)	Product size/bp
HSPA6	NM_002155	5′ TGCAGTCGGACATGAAGCAC 3′ 5′ GTAGCATACGCGCACCTTG 3′	74
ETV4	NM_001986	5′ AGACGGACTTCGCCTACGACT 3′ 5′ TCATAGCCATAGCCCATGGC 3′	112
ANKRD1	NM_014391	5′ TGTCAGACAAGAACAATCCAGATG 3′ 5′ GCTTCCATTAACTTCTCCACAAT 3′	106
REG1A	NM_002909	5′ GACCAGCTCATACTTCATGCTGA 3′ 5′ GGCAATAGAGATCTGCATCAACC 3′	185
CRYBB2	NM_000496	5′ CCAAGATCATCATCTTTGAGCAG 3′ 5′ GATGATCTTGTGCTCTTGGCTG 3′	201
GAPDH	NM_002046	5′ CATCATCCCTGCCTCTACTGG 3′ 5′ GTGGGTGTCGCTGTTGAAGTC 3′	259

### Statistical analysis

The data were analysed by Student’s t-test and are expressed as the mean value±sem. *P* values<0.05 were considered statistically significant.

## Results

### Verification of the yeast bait plasmid pGBKT7-BspC

**Fig. 1.**
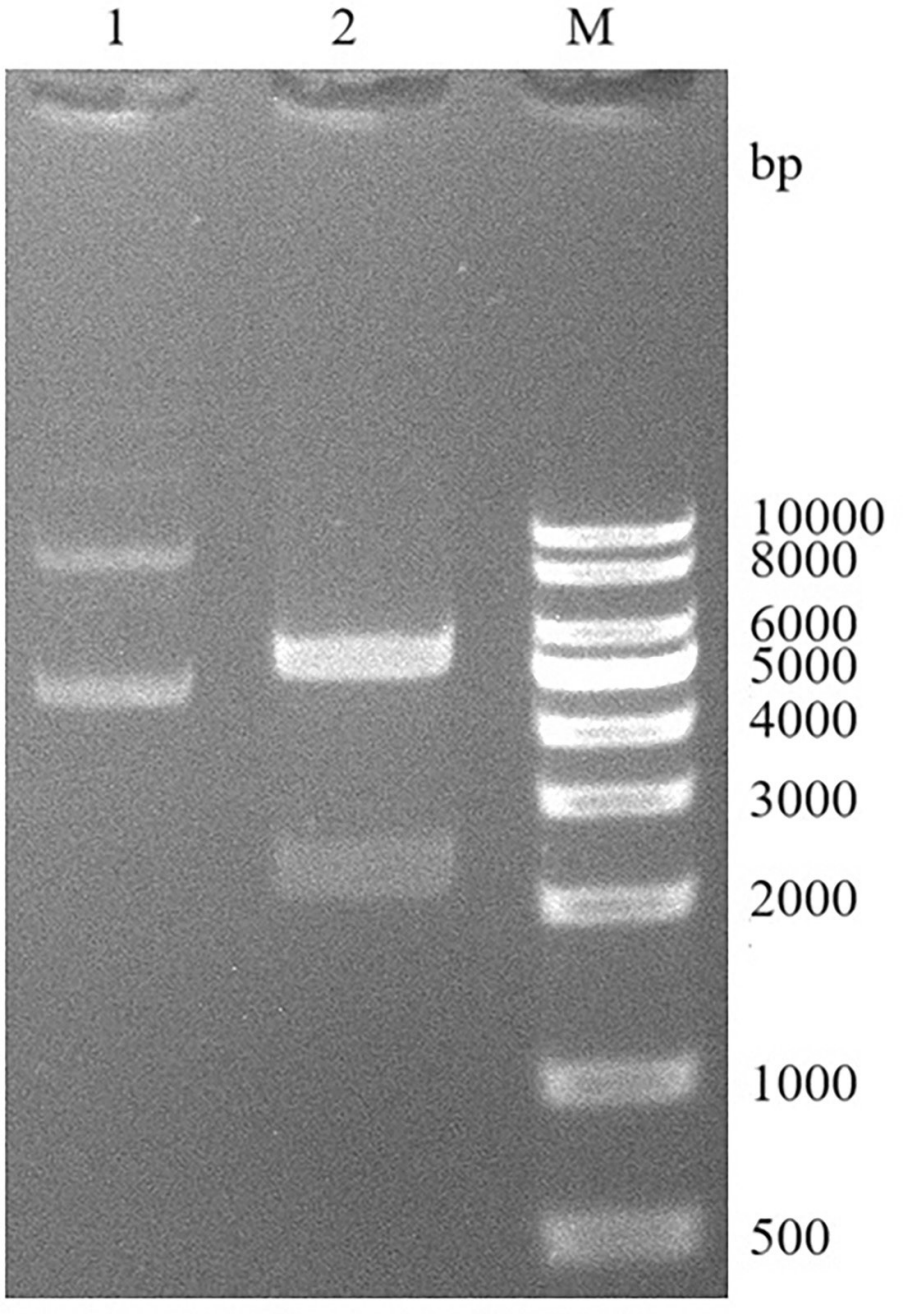
Verification of the yeast bait plasmid pGBKT7-BspC. 1, pGBKT7-BspC plasmid control; 2, double enzyme digestion of the pGBKT7-BspC plasmid; M, DL10000 DNA marker.

EcoRI and BamHI restriction sites were added to the 5′ and 3′ ends of the BspC gene, respectively. The bait recombinant plasmid pGBKT7-BspC was synthesized and constructed by GenScript Biotechnology Co., Ltd. Subsequently, the recombinant plasmid was verified by double digestion with EcoRI and EcoRV. The electrophoresis results revealed the expected bands of ~5,400 bp (containing the BspC gene) and 2,300 bp (Fig. 1). The construction was confirmed to be correct by sequencing.

### Results of self-activation and toxicity assessments of the bait protein BspC

**Fig. 2.**
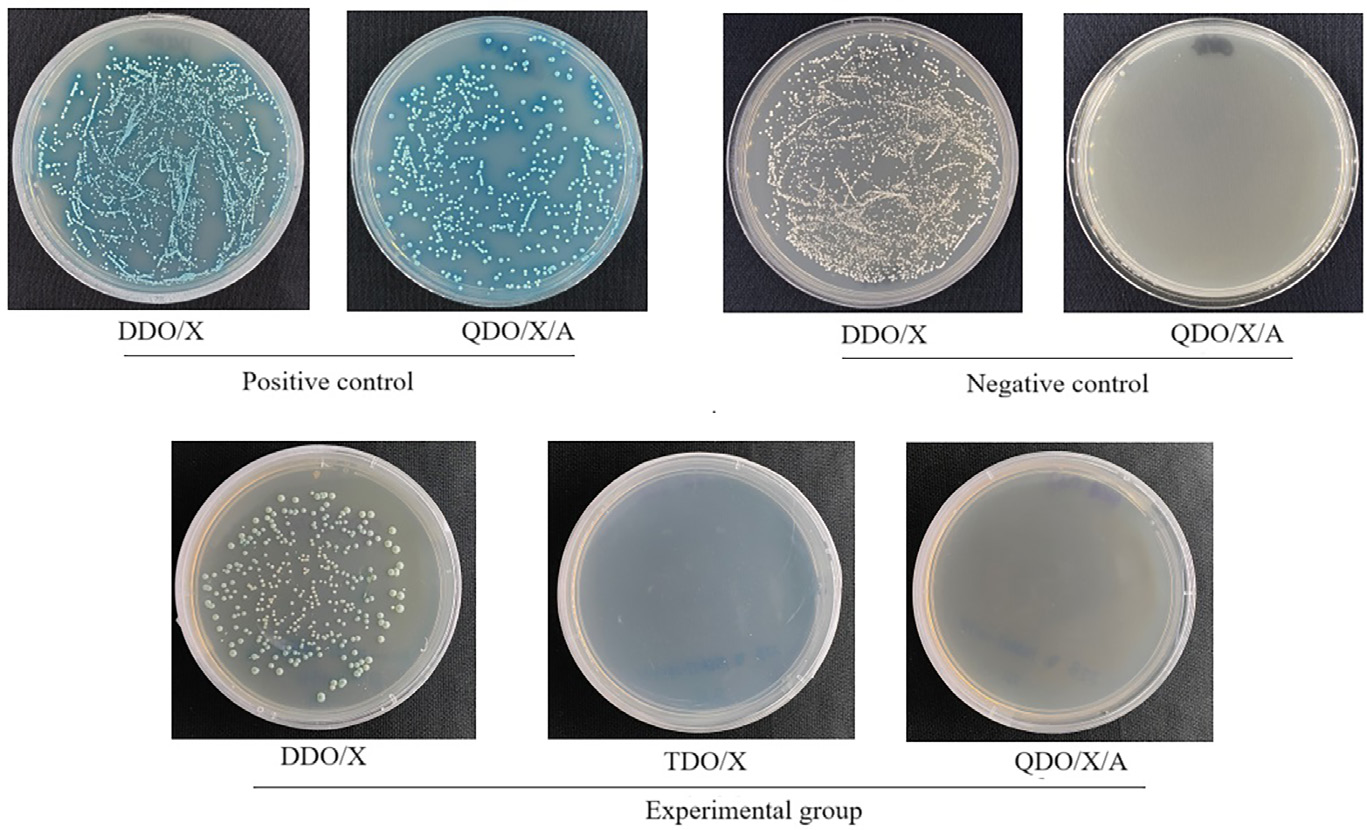
Self-activation and toxicity of the bait plasmid pGBKT7-BspC.

Yeast cells were co-transformed with the bait plasmid pGBKT7-BspC and the prey plasmid pGADT7, alongside positive (pGBKT7-53+pGADT7-T) and negative (pGBKT7-Lam+pGADT7-T) control pairs (see Table 2 for construct details). The transformed cell suspensions were spread on DDO/X, TDO/X and QDO/X/A solid media plates. After 3–5 days, colonies were observed to grow on all the DDO/X plates, indicating that all the transformation systems were successful. The positive control yeast cells grew normally and turned blue on the QDO/X/A plate, whereas no colonies grew on the QDO/X/A plate for the negative control yeast cells, confirming the validity of the positive and negative controls. Yeast cells cotransformed with pGBKT7-BspC and pGADT7 grew blue colonies on DDO/X plates, indicating that the pGBKT7-BspC plasmid was successfully transferred into the yeast cells and expressed the BspC protein. No growth was observed on the TDO/X and QDO/X/A plates, suggesting that the bait protein BspC expressed in the yeast cells had no self-activation activity under these conditions. Moreover, the size and number of colonies formed on the DDO/X plate by this group of yeast cells were essentially the same as those of the negative control yeast cells (Fig. 2), indicating that the bait protein BspC was nontoxic to the yeast cells.

**Table 2.** Summary of yeast two-hybrid constructs used in this study

Construct name	Type/description	Genotypic characteristics (selectable markers)	Phenotypic characteristics on selective media
pGBKT7	Bait vector (BD vector)	Contains the DNA-binding domain (BD) of the Gal4 protein. Selectable marker: TRP1 (allows growth without tryptophan).	Yeast transformed with this vector can grow on synthetic medium lacking tryptophan (SD/-Trp).
pGADT7	Prey vector (AD vector)	Contains the activation domain (AD) of the Gal4 protein. Selectable marker: LEU2 (allows growth without leucine).	Yeast transformed with this vector can grow on synthetic medium lacking leucine (SD/-Leu).
pGBKT7-BspC	Bait construct	pGBKT7 vector with the gene of interest (BspC) inserted. Selectable marker: TRP1.	Yeast can grow on SD/-Trp. The bait protein (BD-BspC) is expressed.
pGADT7-Library	Prey library construct	pGADT7 vectors containing cDNA fragments from a target organism/tissue (e.g. *M. oryzae* cDNA library). Selectable marker: LEU2.	Yeast containing these plasmids can grow on SD/-Leu. A vast number of potential ‘prey’ proteins (AD-fusions) are expressed.
pGBKT7-53	Positive control bait	pGBKT7 vector with the well-known murine p53 gene. Selectable marker: TRP1.	Yeast can grow on SD/-Trp.
pGADT7-T	Positive control prey	pGADT7 vector with the SV40 large T-antigen gene, which is known to interact with p53. Selectable marker: LEU2.	Yeast can grow on SD/-Leu.
pGBKT7-Lam	Negative control bait	pGBKT7 vector with the human lamin C gene, which is not expected to interact with most proteins. Selectable marker: TRP1.	Yeast can grow on SD/-Trp.

### Screening of proteins that interacted with BspC

The pGBKT7-BspC plasmid and the RAW264.7 cell cDNA library were cotransformed into competent cells of the yeast strain Y2H Gold. The transformed cell suspension was spread on TDO/X plates and incubated for 3–5 days. The number of single colonies was counted to initially screen for host proteins interacting with BspC. Seven blue colonies were obtained. These blue colonies were then transferred to QDO/X/A selection plates for rescreening, and five colonies turned blue. The five positive colonies were cultured in QDO liquid medium at 30 °C for 24 h, after which the plasmids were extracted and sequenced. The sequences were analysed by BLASTX in the NCBI database, and four potential host proteins that could interact with BspC were identified. The corresponding amino acid sequences encode the proteins FDX1, DNAJA1, HSPA5 and PTPN2 (Table 3). These findings indicated that a total of four host proteins that interacted with BspC were identified after two rounds of screening.

**Table 3.** Information of host proteins with potential interaction with BspC

ID	Host protein information	GenBank login no.	Identity
1	Mus musculus ferredoxin 1 (FDX1)	NM_007996.2	100%
2	Mus musculus DnaJ heat shock protein family (Hsp40) member A1 (DNAJA1)	NM_001164671.2	100%
3	Mus musculus heat shock protein 5 (HSPA5)	NM_001163434.1	100%
4	Mus musculus protein tyrosine phosphatase, non-receptor type 2 (PTPN2)	NM_008977.3	100%

### Results of the cross-verification experiment for the proteins that interacted with BspC

**Fig. 3.**
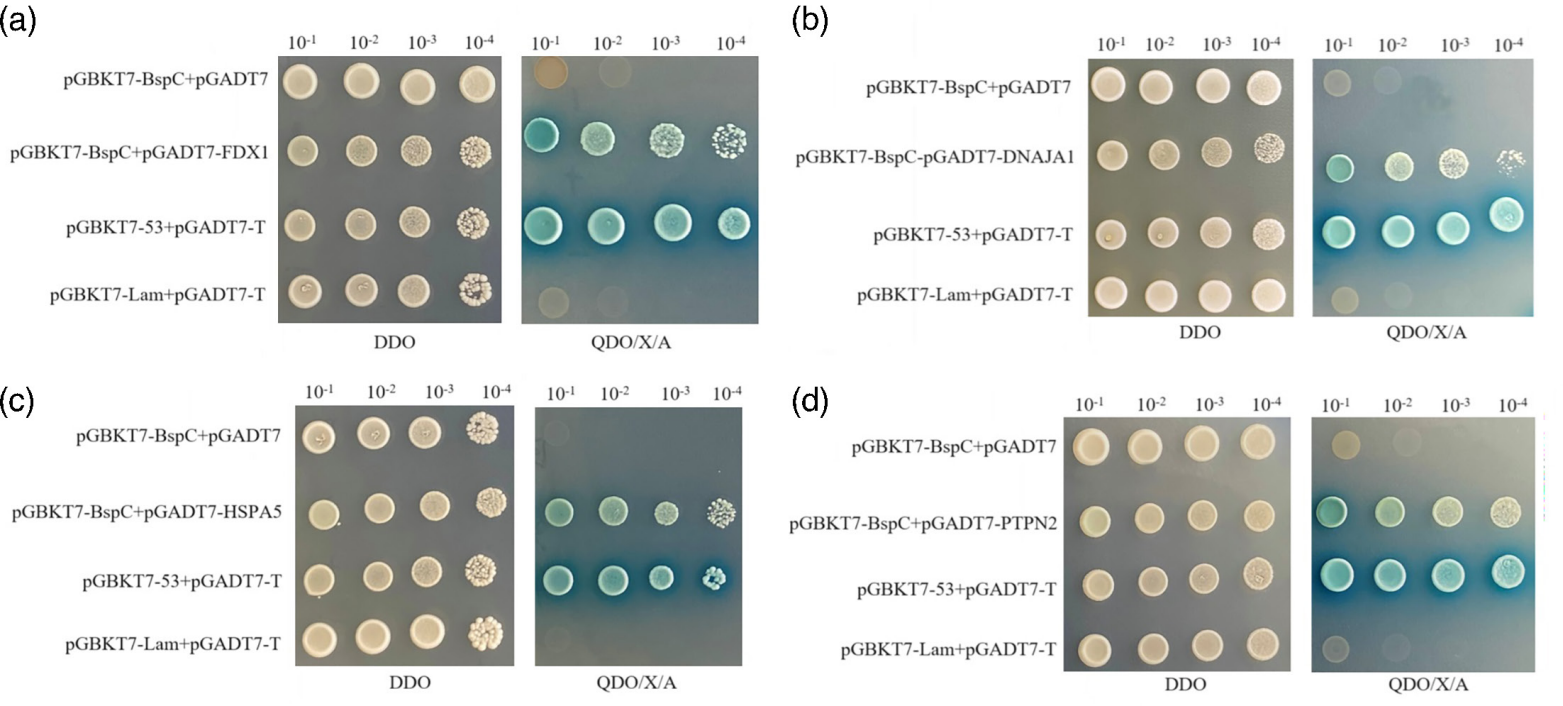
Cross-verification test of BspC-interacting proteins.

The four plasmids with high homology to the genes of the host proteins that interacted with BspC, namely, pGADT7-FDX1, pGADT7-DNAJA1, pGADT7-HSPA5 and pGADT7-PTPN2, were cotransformed with pGBKT7-BspC into Y2H Gold competent yeast cells. Negative and positive control groups and a blank plasmid control group were set up. The transformed yeast cultures were spread on DDO and QDO/X/A plates and incubated at 30 °C for 3–5 days. Blue single colonies were picked and resuspended in 0.9% NaCl solution, then 10-fold serially diluted and spread on DDO and QDO/X/A plates. After 3–5 days of incubation, the growth of the yeast in each group was observed. The results revealed that the positive control group, negative control group and blank plasmid control group all grew normally on the DDO plates. The positive control group produced blue colonies on the QDO/X/A plates, whereas the negative control group and blank plasmid control group did not grow on the QDO/X/A plates, indicating that the experimental system was set up correctly. All experimental groups had colonies growing and turning blue on the QDO/X/A plates. Yeast transformed with different dilutions of pGBKT7-BspC+pGADT7-FDX1, pGBKT7-BspC+pGADT7-DNAJA1, pGBKT7-BspC+pGADT7-HSPA5 and pGBKT7-BspC+pGADT7-PTPN2 produced blue colonies on the QDO/X/A plates, but the number of colonies decreased gradually with increasing dilution, which was basically consistent with the results of the positive control (Fig. 3). This finding further indicated that BspC interacted with the host proteins FDX1, DNAJA1, HSPA5 and PTPN2.

### Construction and assessment of the recombinant pTT5-BspC plasmid

**Fig. 4.**
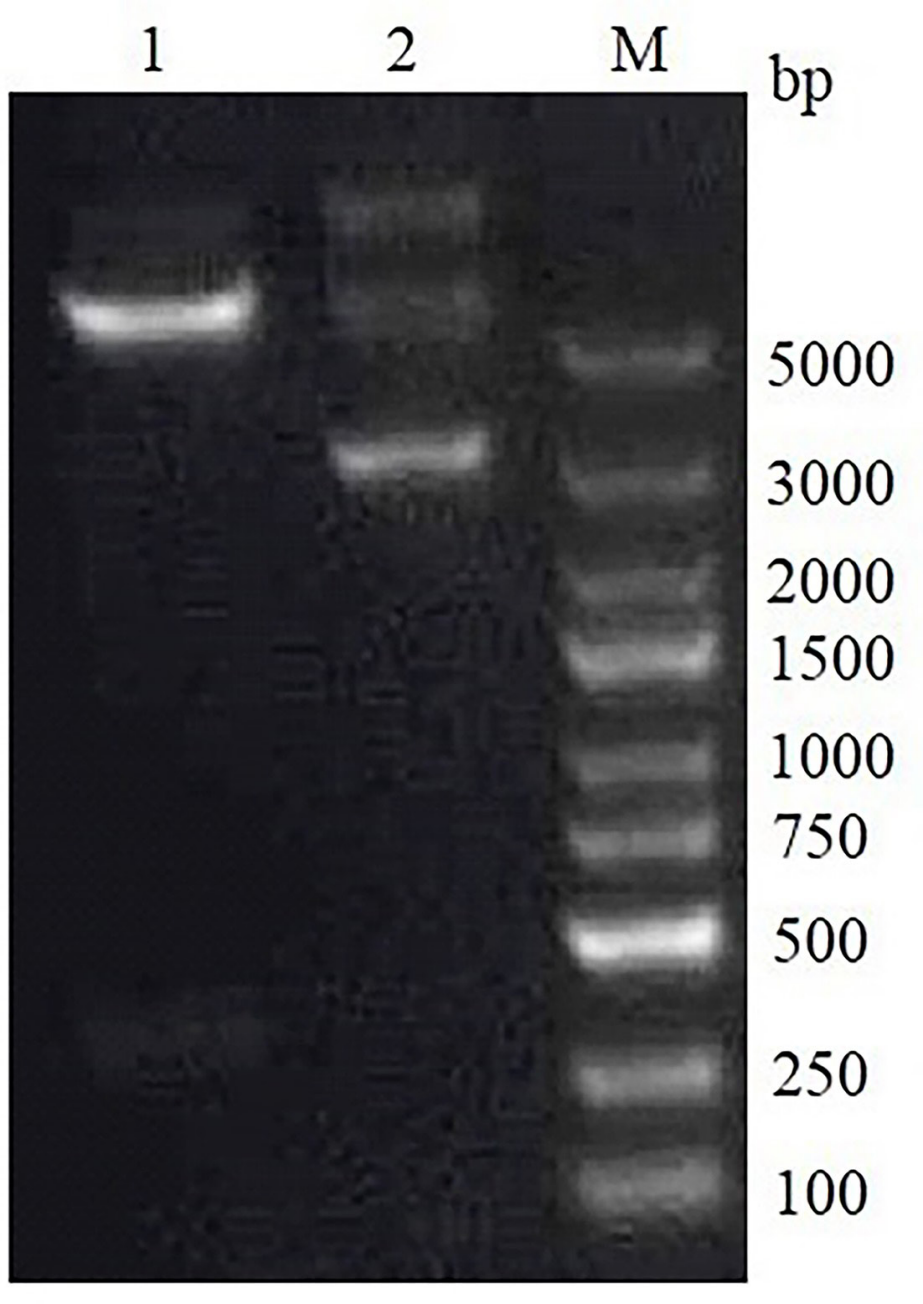
Identification of the double enzyme digestion of the recombinant plasmid pTT5-BspC; 1, double enzyme digestion of the recombinant plasmid pTT5-BspC; 2, enzyme digestion of the empty plasmid pTT5-BspC; M, DL 5000 DNA molecular weight standard.

The BspC gene sequence was retrieved from NCBI, and restriction sites (EcoRI/HindIII) were added. The BspC gene sequence was synthesized by General Biosystems (Anhui) Co., Ltd. (China) and then cloned and inserted into the eukaryotic expression vector pTT5. DH5α competent cells were transformed, and positive clones were picked. The target fragment of ~459 bp was verified by EcoRI/HindIII digestion (Fig. 4). The sequencing results of the plasmid revealed that the correct BspC sequence was inserted, indicating the successful construction of the recombinant plasmid, which was designated pTT5-BspC.

### Assessment of BspC protein expression in cells

**Fig. 5.**
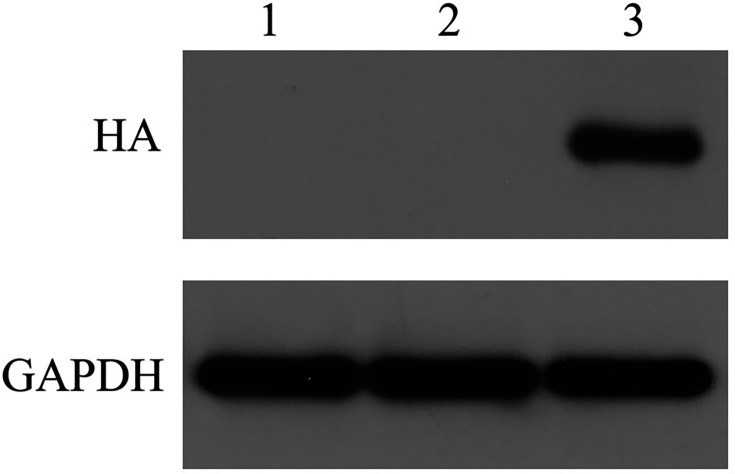
Assessment of BspC protein expression in cells.

After total protein was extracted from HEK293T cells transfected with pTT5-BspC, the protein expression of BspC was detected by Western blotting. The results revealed that the HA antibody detected the target band at ~18 kDa, which was consistent with the theoretical value of the sum of the sizes of the BspC and HA proteins (Fig. 5). The results indicated that the cells transfected with pTT5-BspC efficiently expressed the BspC protein.

### Assessment of apoptosis-related protein expression

**Fig. 6.**
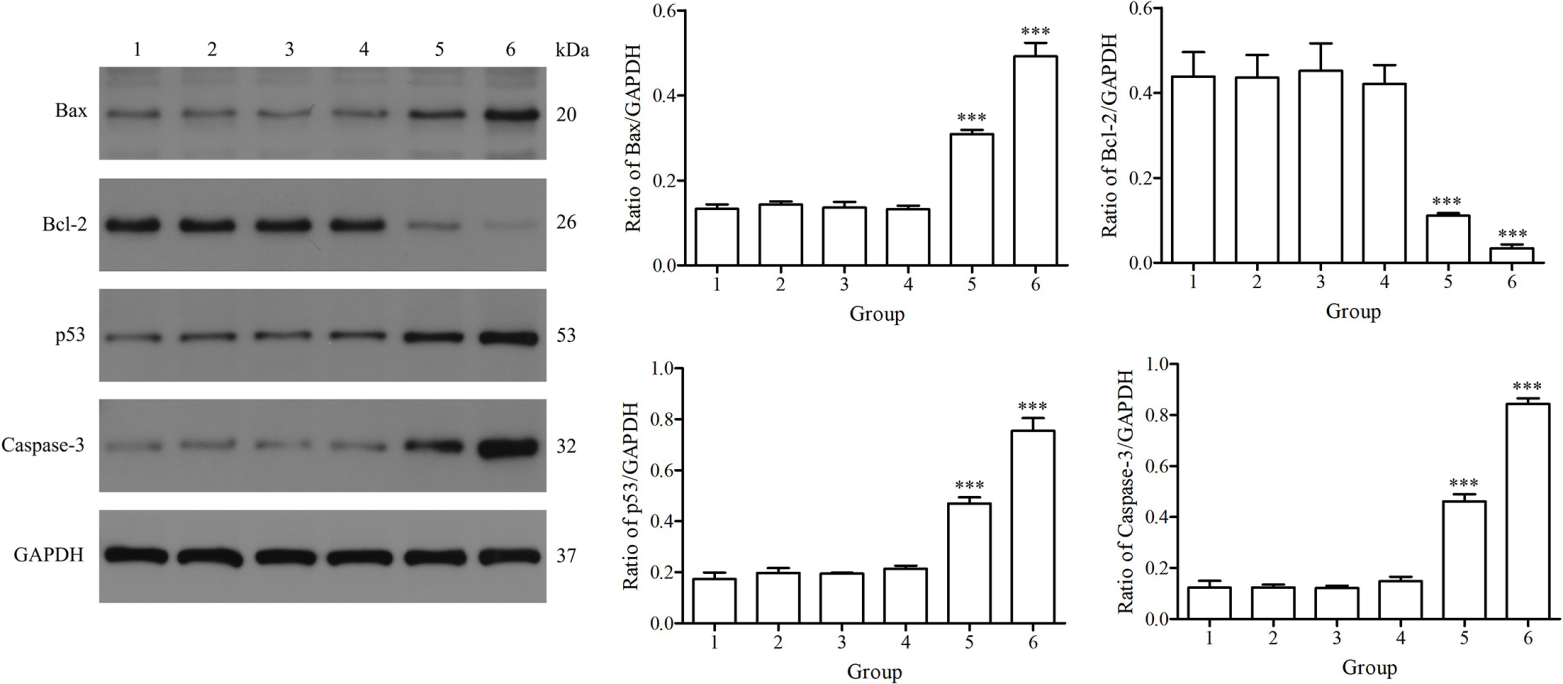
Changes in the expression of apoptosis-related proteins.

Total protein was extracted from HEK293T cells transfected with pTT5-BspC, and the expression levels of Bax, Bcl-2, p53 and Caspase-3 were assessed by Western blotting. Compared with that in the pTT5 group, the expression of the apoptosis-related proteins Bax, p53 and Caspase-3 in cells transfected with pTT5-BspC was significantly upregulated at 24 and 48 h, whereas the expression of the antiapoptotic protein Bcl-2 was significantly downregulated (Fig. 6). These results indicated that the BspC protein promoted the expression of apoptosis-related proteins.

### Effects of the BspC protein on the percentage of apoptotic cells

**Fig. 7.**
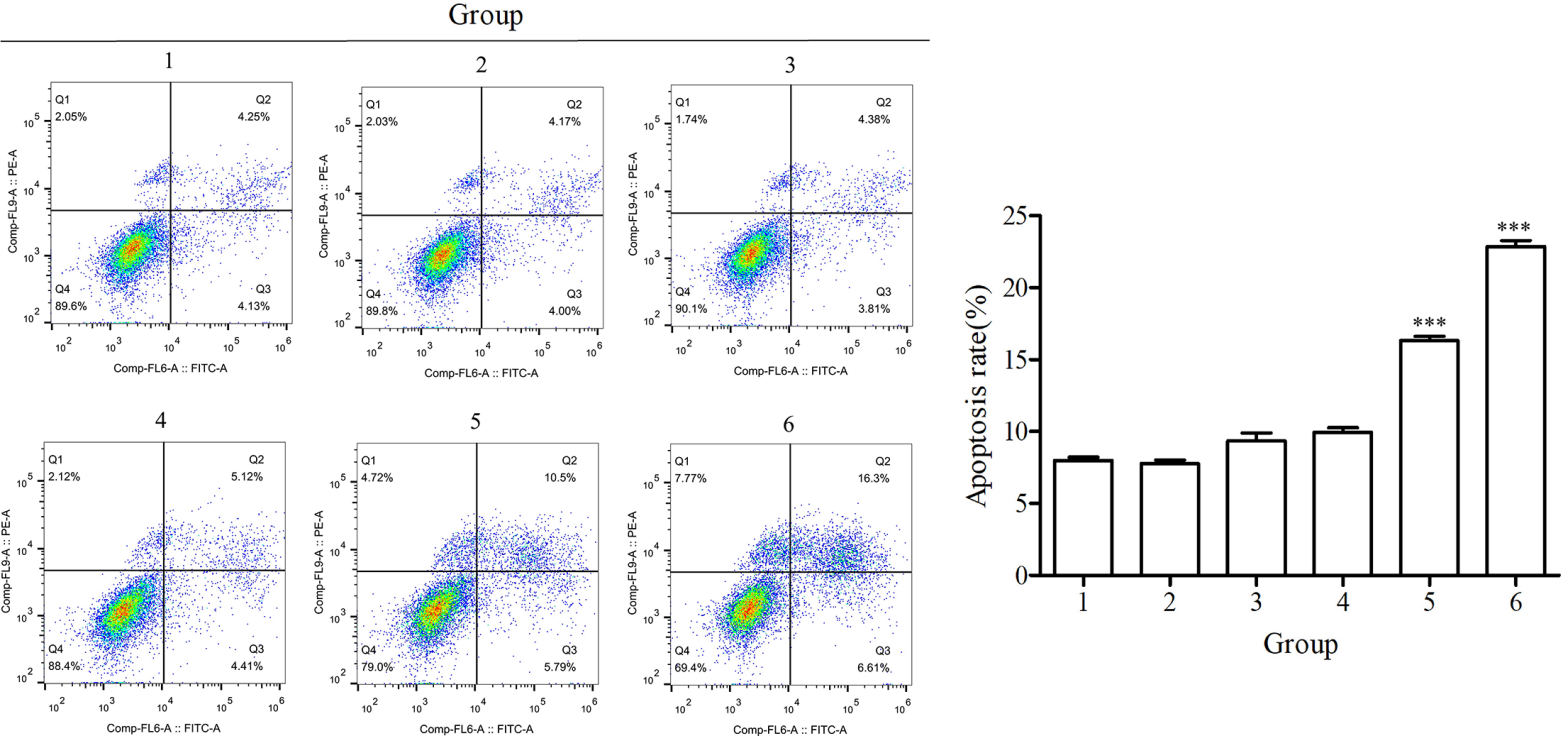
Effects of the BspC protein on cell apoptosis. 1, normal culture 24 h cell group; 2, normal culture 48 h cell group; 3, pTT5 plasmid transfection 24 h cell group; 4, pTT5 plasmid transfection 48 h cell group; 5, pTT5-BspC plasmid transfection 24 h cell group; 6, pTT5-BspC plasmid transfection 48 h cell group. ***P<0.001.

The effects of BspC protein expression in HEK293T cells on the percentage of apoptotic cells were assessed. The results revealed that at 24 and 48 h, the percentages of apoptotic cells in the pTT5-BspC group were 16.32±0.30% and 22.86±0.30 %, respectively, whereas those in the pTT5 control group were 9.33±0.57% and 9.96±0.30 %, respectively. Therefore, compared with those of the pTT5 control group, the percentage of apoptotic cells in the pTT5-BspC group at 24 and 48 h was significantly greater (*P*<0.001) (Fig. 7). These results indicated that the BspC protein promoted apoptosis in the cells.

### Laser scanning confocal microscopy observations

**Fig. 8.**
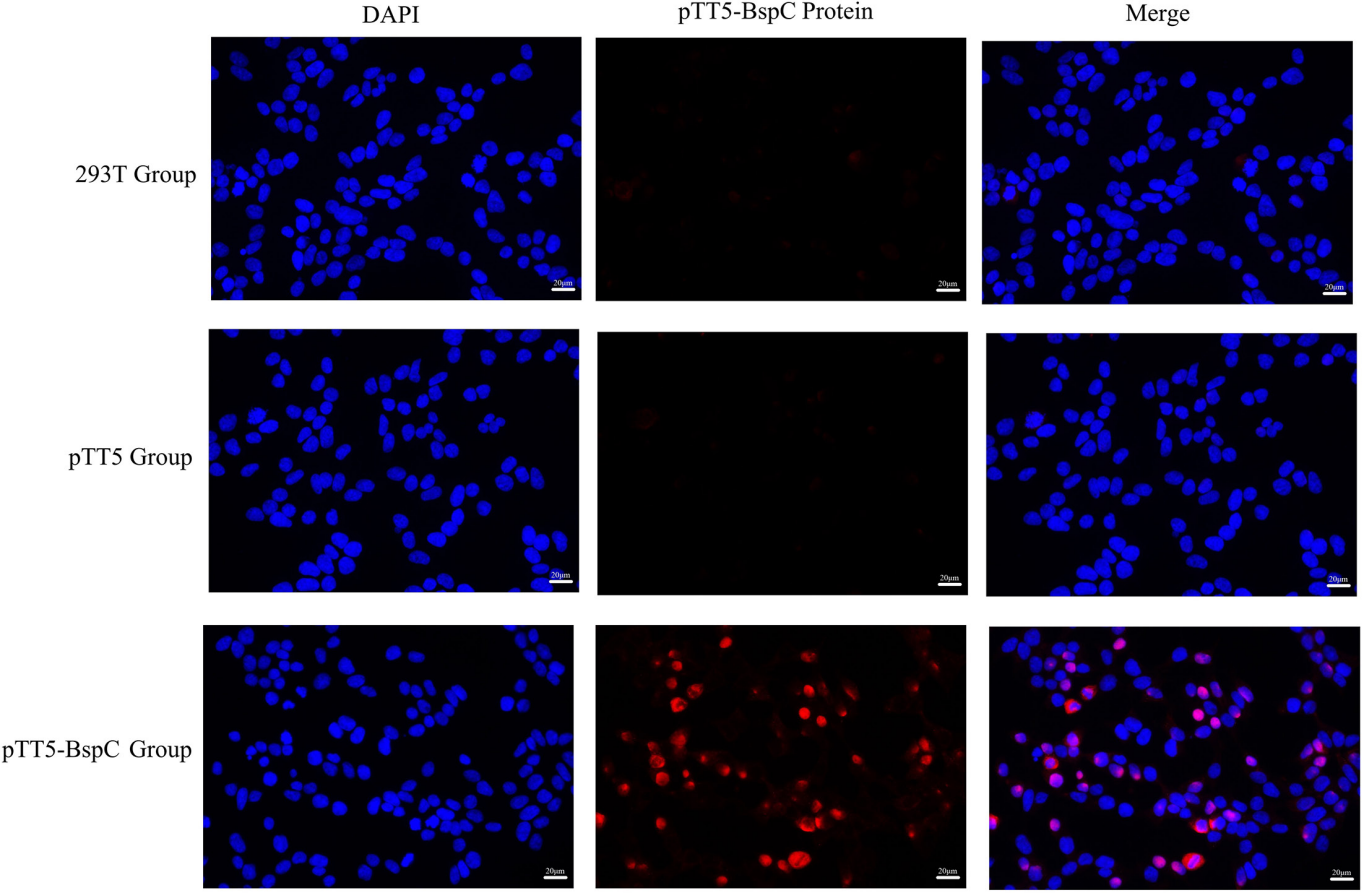
Localization of the BspC protein observed by laser scanning confocal microscopy.

To detect the subcellular localization of the BspC protein, fluorescence staining analysis was performed on cells transfected with the recombinant plasmid pTT5-BspC. The results revealed that the pTT5-BspC fusion protein presented red fluorescence in the cells, whereas the cell nuclei stained with DAPI presented blue fluorescence. The two fluorescence signals did not completely overlap (Fig. 8), indicating that the pTT5-BspC fusion protein was located in both the cytoplasm and the nucleus.

### Effects of the BspC protein on cytokine secretion

**Fig. 9.**
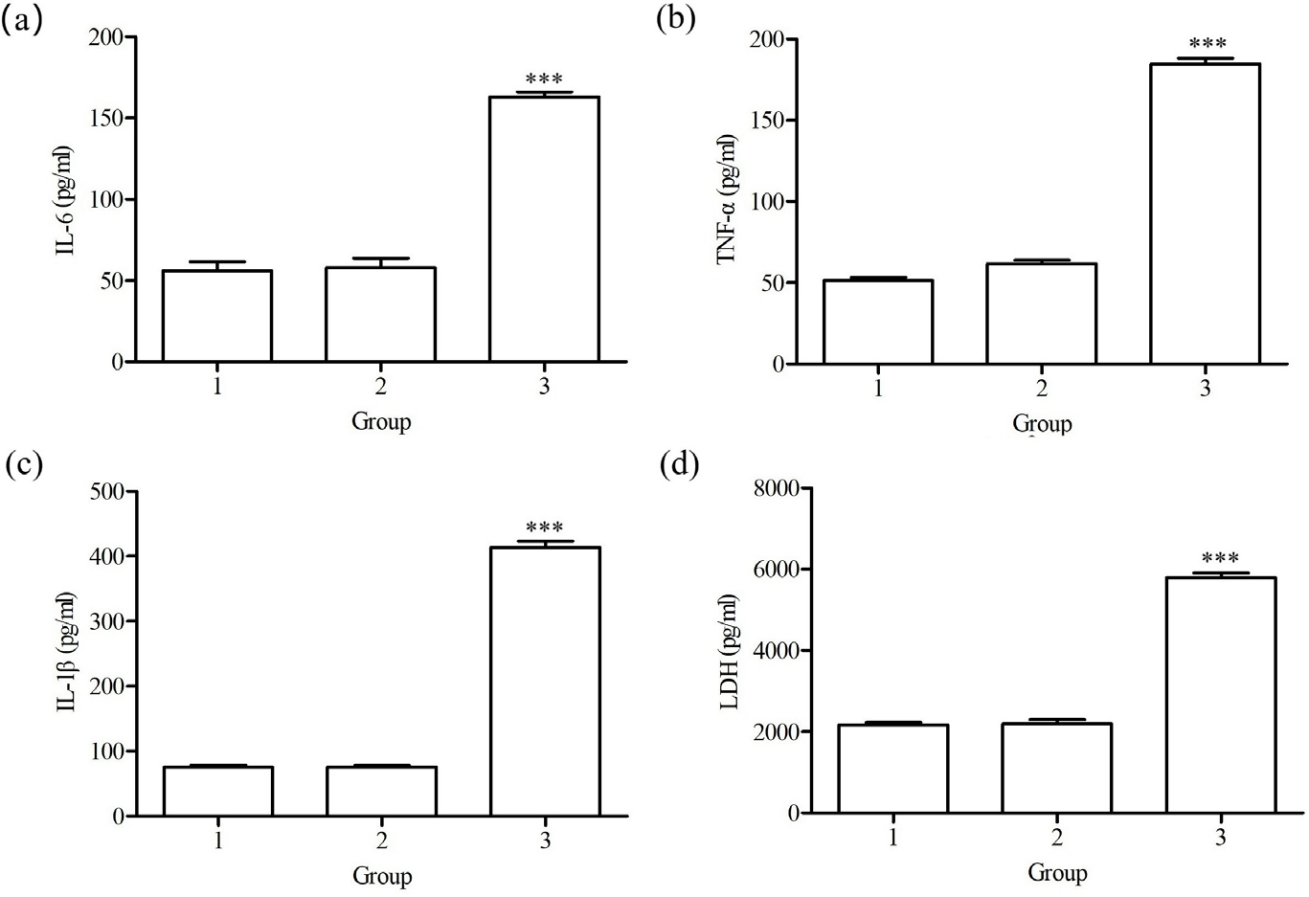
Effects of the BspC protein on cytokine levels. 1, normal culture for 48 h cell group; 2, transfection with pTT5 plasmid for 48 h cell group; 3, transfection with pTT5-BspC plasmid for 48 h cell group. (a) changes in IL-6 levels; (b) changes in TNF-α levels; (c) changes in IL-1β levels; (d) changes in LDH levels. ***P<0.001.

The effects of intracellularly expressed BspC protein on cytokines were assessed. The results revealed that the levels of IL-6, TNF-α, IL-1β and LDH secreted by the cells in the pTT5-BspC transfection group at 48 h were significantly greater than those in the pTT5 empty plasmid group (*P*<0.001, Fig. 9). These results indicated that the BspC protein increased levels of cellular immune factors and promoted the secretion of proinflammatory cytokines.

### Effects of the BspC protein on biochemical indicators in cells

**Fig. 10.**
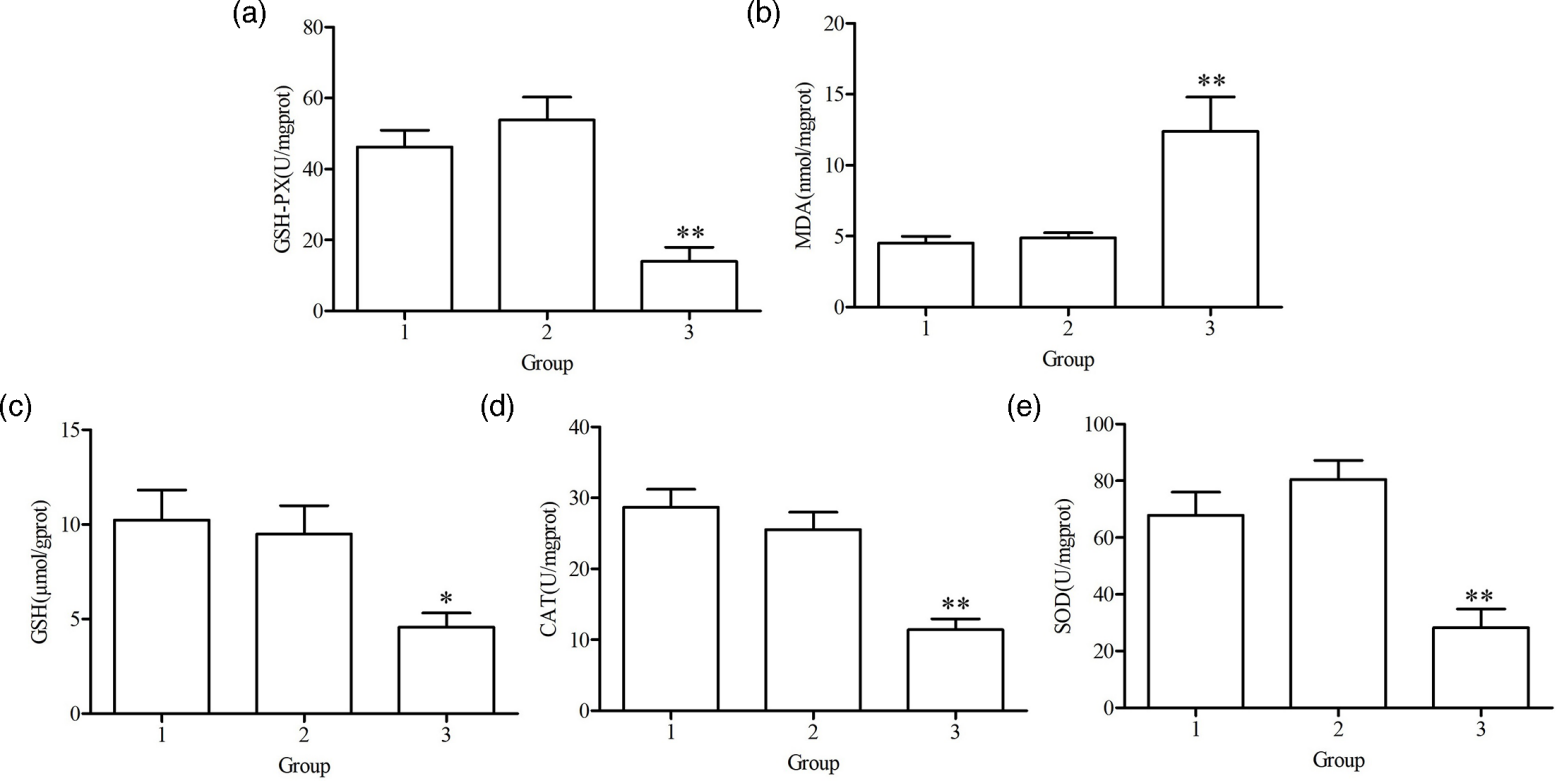
Effects of BspC protein expression on biochemical indicators. 1, normal culture for 48 h cell group; 2, transfection with the pTT5 plasmid for 48 h cell group; 3, transfection with the pTT5-BspC-HA plasmid for 48 h cell group. (a) Changes in GSH-PX activity; (b) changes in MDA activity; (c) changes in GSH activity; (d) changes in CAT activity; (e) changes in SOD activity. *P<0.05, **P<0.01 and ***P<0.001.

The effects of BspC protein expression in cells on biochemical indicators were detected. Compared with those in the pTT5 empty plasmid group cells, the activities of GSH-PX, GSH, CAT and SOD in the pTT5-BspC-transfected 48 h group of cells were significantly lower, whereas the activity of MDA was significantly greater (Fig. 10). These results indicated that the BspC protein inhibited the antioxidant capacity of host cells.

### Transcriptomic analysis of BspC protein-stimulated host cells screening of DEGs

**Fig. 11.**
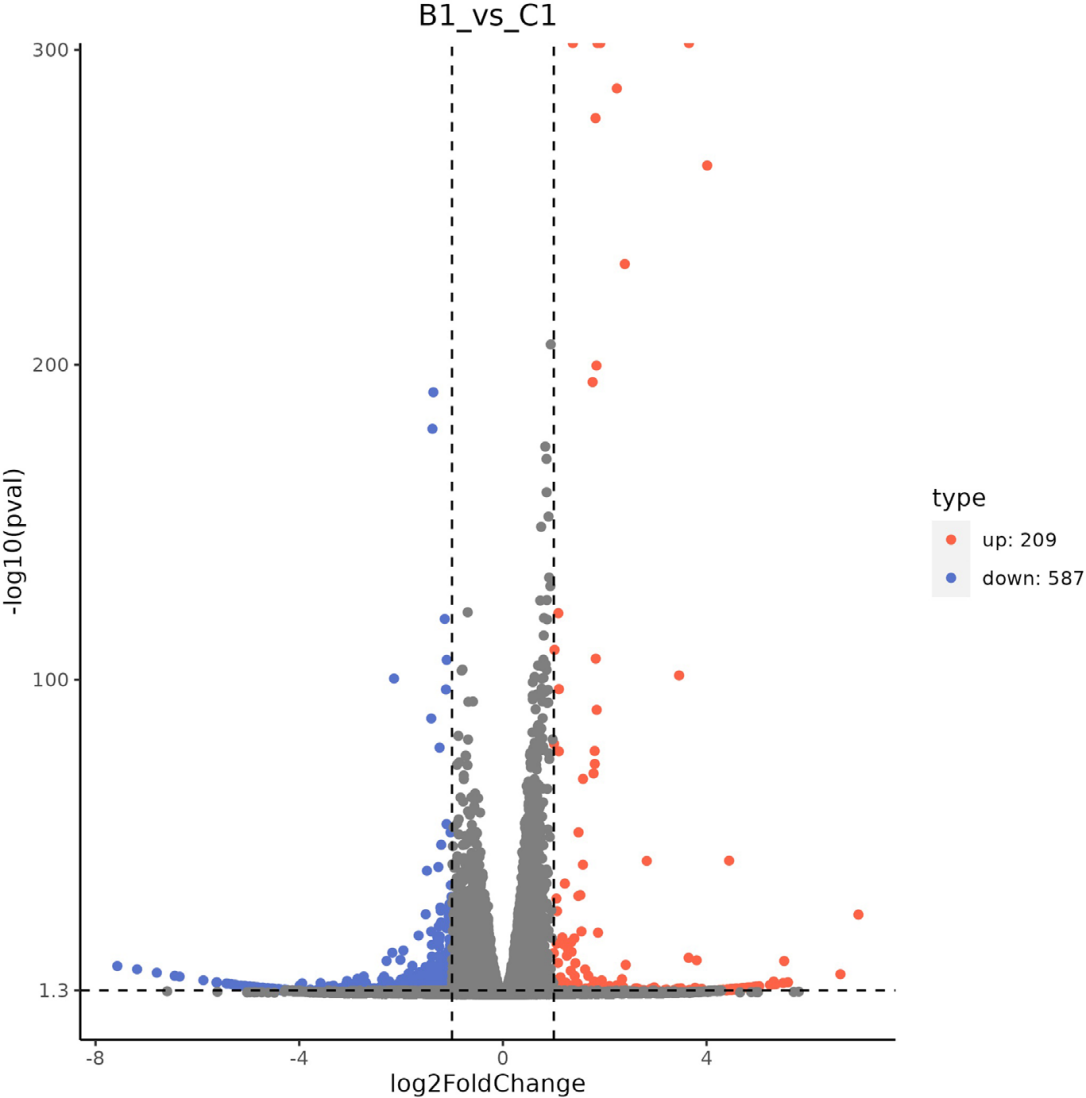
Volcano plot for differential analysis.

Differential analysis of the expression profile data of the experimental group and the control group revealed a total of 796 DEGs, including 209 upregulated genes and 587 downregulated genes (Fig. 11).

### GO functional enrichment analysis of DEGs

**Fig. 12.**
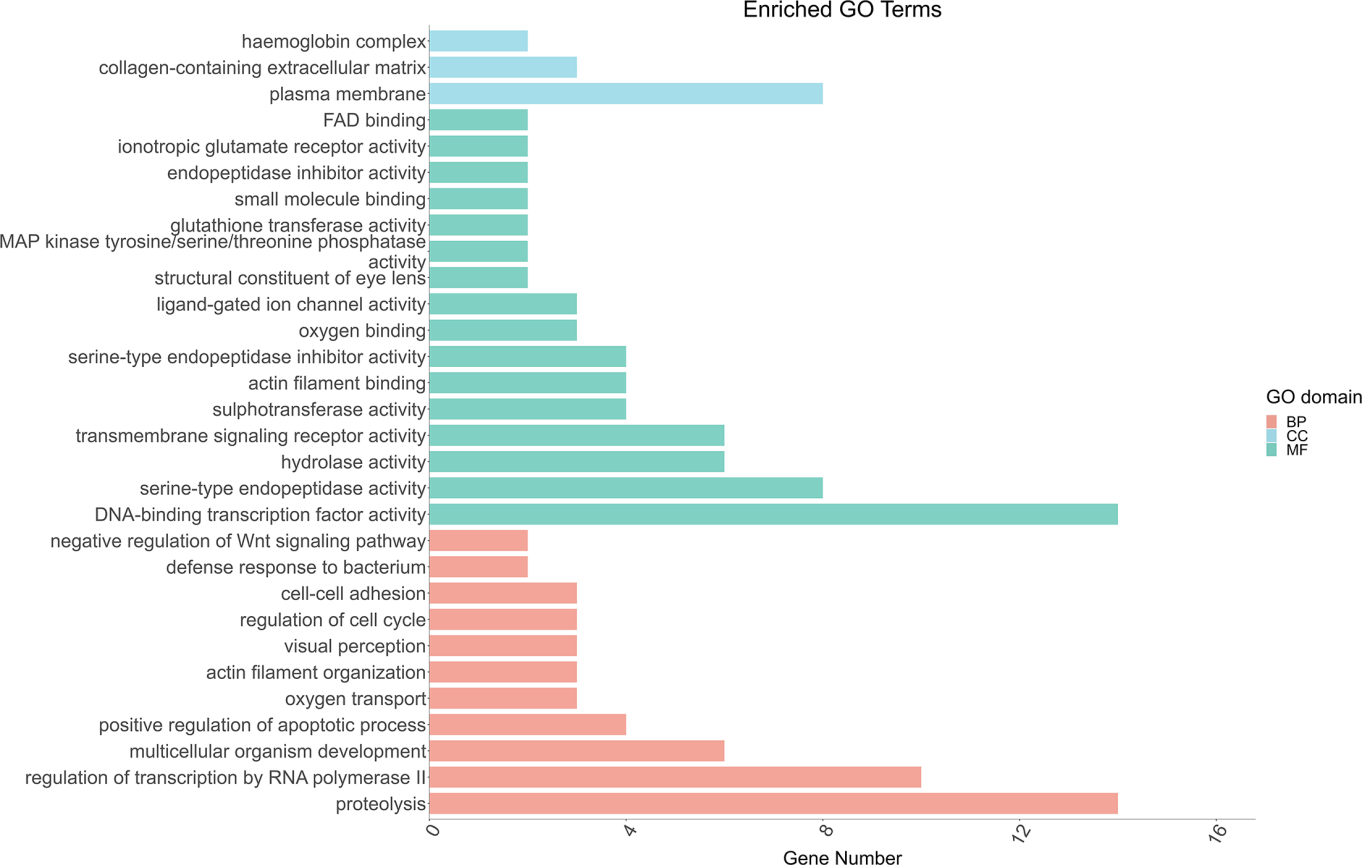
Statistical analysis chart of the GO enrichment analysis annotation.

GO functional enrichment analysis was conducted on the DEGs. The results revealed that 796 DEGs were classified and annotated in three major categories: biological process (BP), cellular component (CC) and molecular function (MF). The main biological functions of the DEGs in the BP category were positive regulation of the apoptotic process, oxygen transport and regulation of transcription by RNA polymerase II; in the CC category, the DEGs were associated mainly with the haemoglobin complex, plasma membrane and extracellular region; and in the MF category, the DEGs were associated mainly with serine-type endopeptidase activity, transmembrane signalling receptor activity and DNA-binding transcription factor activity (Fig. 12).

### KEGG pathway enrichment analysis of DEGs

**Fig. 13.**
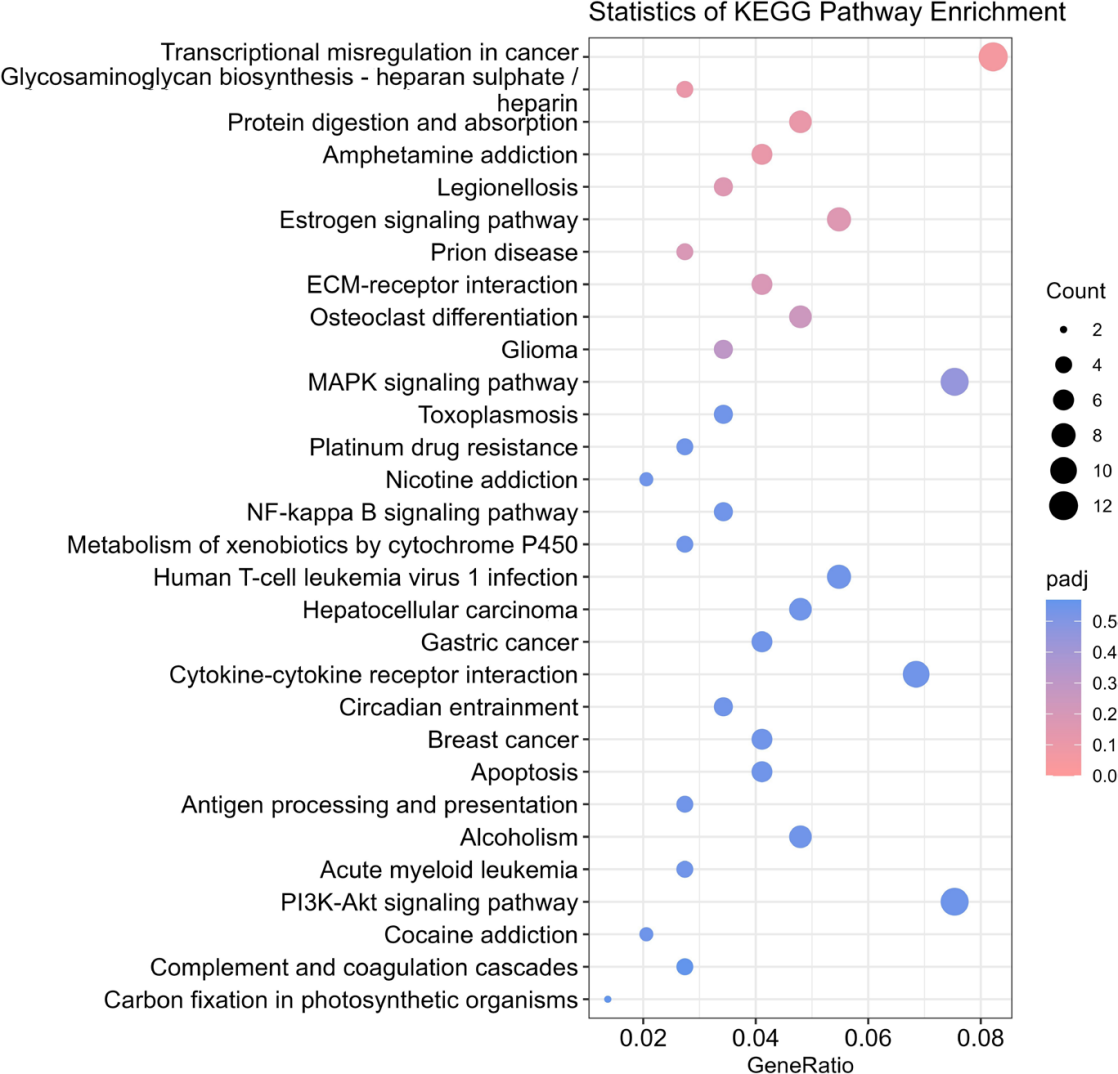
Scatter plot of KEGG pathway enrichment of DEGs.

KEGG pathway enrichment analysis was conducted on the DEGs. The results revealed that the DEGs were enriched mainly in signalling pathways such as the ECM-receptor interaction, the MAPK signalling pathway, the NF-κB signalling pathway and the apoptosis pathway (Fig. 13).

### qRT-PCR verification

**Fig. 14.**
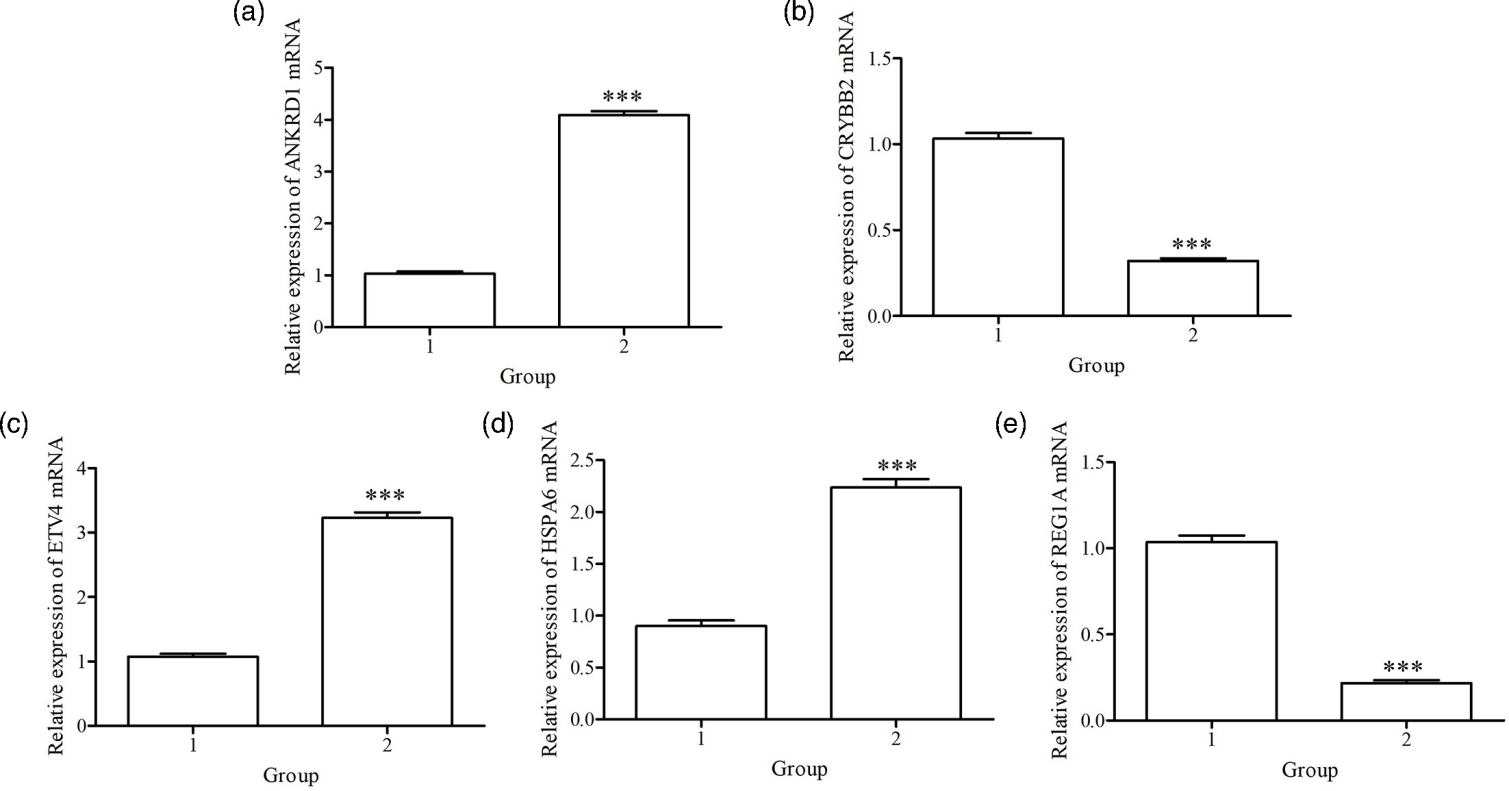
qRT-PCR verification of transcriptome data results. 1, Cells transfected with the pTT5 plasmid for 48 h; 2, cells transfected with the pTT5-BspC plasmid for 48 h. (a) Changes in the expression of ANKRD1; (b) changes in the expression of CRYBB2; (c) changes in the expression of EVT4; (d) changes in the expression of HSPA6; (e) changes in the expression of REG1A. ***P<0.001.

Five DEGs were randomly selected for qRT-PCR verification. Among the five DEGs, three were upregulated, and two were downregulated (Fig. 14). The experimental results were consistent with the transcriptome sequencing results, further confirming the reliability of the transcriptome sequencing data.

## Discussion

The two effector protein genes, VceC and VceA, share the upstream coding sequence of the virB promoter region and are coregulated by VjbR for their transcription [14]. Studies have shown that compared with the wild-type *B. abortus* strain, a VceC deletion strain can promote its proliferation within cells and induce proinflammatory responses within cells [15]. The *Brucella* VceC deletion strain can induce apoptosis through the endoplasmic reticulum stress pathway. Infection of goat embryo trophoblast cells by the *Brucella* VceC deletion strain can induce endoplasmic reticulum stress responses, and different endoplasmic reticulum stress states can affect the proliferation of the VceC deletion strain within goat embryo trophoblast cells [10]. Research has shown that the *B. abortus* VceA gene deletion strain can inhibit the apoptosis of human embryo trophoblast cells [11]. The Toll receptor domain of the BtpB effector protein is present in eukaryotic Toll-like proteins and their downstream Toll-like adaptor proteins. Their interactions can cause the nuclear translocation of NF-κB and the production of proinflammatory cytokines and type I interferons [16]. BPE123 is an effector protein transported by *Brucella* through VirB. Studies have shown that human α-enolase interacts with BPE123 and that the degradation of α-enolase can inhibit the proliferation of *B. abortus* within human cervical cancer cells, indicating that α-enolase plays a role in *Brucella* infection of cells [17]. Through yeast two-hybrid and coimmunoprecipitation, the host proteins nucleoside diphosphate kinase 2 and creatine kinase B that interacted with the *Brucella*-secreted protein BspJ were identified, and the BspJ gene deletion strain was shown to induce the apoptosis of macrophages and inhibit the proliferation of *Brucella* within host cells [12]. The effector proteins BspA, BspB and BspF can inhibit the normal protein secretion function of host cells and promote the biosynthesis of replicative BCV within cells and the proliferation of *Brucella*. Virulence evaluation revealed that the bacterial load of the BspF deletion strain decreased both in cells and in the spleens of infected mice. BspC and BspE are located around the cell nucleus, with BspC located in the Golgi apparatus and BspE forming vesicles [8]. BspB interacts with the oligomeric Golgi tethering complex of host cells, hijacking the transport of Golgi vesicles, allowing BCV to move stably towards the endoplasmic reticulum and promoting the biosynthesis of replicative BCV and the intracellular replication of *Brucella* [18]. Notably, our findings demonstrate that BspC promotes apoptosis and proinflammatory responses, which contrasts with the anti-apoptotic roles of effectors like VceA and the intracellular replication promotion by the Bsp family proteins (BspA, B, F). This functional divergence suggests that *Brucella* employs a sophisticated temporal or spatial regulation of its effectors to finely balance host cell death and survival, ultimately facilitating persistent infection. Specifically, BspC may function in concert with other effectors such as BtpB in modulating NF-κB signalling, albeit towards different outcomes, or it may act early to counteract host defenses through inflammation and apoptosis, thereby creating a niche subsequently exploited by pro-replication effectors like BspB. Our study revealed that the BspC protein can promote the expression of the apoptosis-related proteins Bax, p53 and Caspase-3 while inhibiting the expression of the antiapoptotic protein Bcl-2, increasing the percentage of apoptotic cells. Therefore, the BspC protein promotes cell apoptosis. The yeast two-hybrid system is a system for screening and identifying protein‒protein interactions within cells on the basis of transcription factors and has been widely used in the study of virus‒host interactions [19,20]. In this study, the BspC gene was cloned and inserted into the yeast two-hybrid expression vector pGBKT7, and BspC was used as the bait protein to preliminarily identify host proteins that interact with BspC from the cDNA library of the mouse macrophage cell line RAW264.7. After successfully constructing the BspC bait plasmid and the prey library from RAW264.7 cells, five positive clones were obtained. Sequencing was subsequently performed, and the sequencing results were analysed with NCBI blast. Four potential interacting host proteins were identified, and verification of their cointeraction was conducted, which initially revealed that BspC interacted with the FDX1, DNAJA1, HSPA5 and PTPN2 proteins, laying a foundation for further research on the biological functions of BspC and its involvement in *Brucella* pathogenicity.

TNF, which is also induced by *B. abortus* infection, was recently demonstrated to play a crucial role in inducing anti-*Brucella* effectors by regulating the function of nuclear factor kappa-light-chain-enhancer of activated B cells (NF-κB) in macrophages [21]. The proinflammatory cytokine IL-6 was initially characterized as an inducer of B-cell growth and antibody production; however, IL-6 has been implicated in other immunological processes, including CD4+ T cell differentiation or proliferation and the function of cytotoxic CD8+ T cells [22,23]. When the cell membrane is damaged or its permeability increases, LDH can leak out into the extracellular space, resulting in a significant increase in the activity of LDH in the extracellular fluid. Therefore, the activity of LDH in the cell culture supernatant can reflect the degree of cell membrane damage, and a change in membrane permeability is a common early response when many toxic substances act on the cell membrane and indicates the degree of cell damage. The BspC protein can promote the secretion of IL-6, TNF-α, IL-1β and LDH by cells. These results indicated that the BspC protein affected the levels of cellular immune factors and promoted the secretion of proinflammatory cytokines. This pro-inflammatory role of BspC is particularly intriguing in the context of other *Brucella* effectors. For instance, while BtpB also engages NF-κB pathways, its outcome can be context-dependent. The net effect of BspC-induced inflammation might be to divert or modulate the immune response in a way that, paradoxically, benefits bacterial establishment, perhaps by attracting and eliminating susceptible immune cells or by contributing to the granulomatous pathology characteristic of brucellosis. Currently, there are no reports on the differences in the transcriptome expression profiles of HEK293T cells stimulated with the BspC protein. To explore the changes in the gene expression profile of HEK293T cells after treatment with the BspC protein, the eukaryotic expression plasmid containing the BspC protein was transfected into HEK293T cells for 48 h, and the total RNA of the experimental group and the control group was extracted for transcriptome sequencing. A comparison of the control group and the experimental group revealed a total of 796 DEGs, including 209 upregulated genes and 587 downregulated genes. GO and KEGG enrichment analyses of the DEGs revealed that most of the DEGs were enriched in signalling pathways such as the MAPK signalling pathway, NF-κB signalling pathway and apoptosis pathway. These pathways may mediate the process by which the BspC protein promotes cell apoptosis and cytokine secretion.

MDA, a representative product of lipid peroxidation in cells, is a marker of oxidative damage, and its production is an indirect indicator of the amount of reactive oxygen species generated in the body [24]; a decrease in intracellular GSH levels can increase the sensitivity of cells to harmful stimuli [25], while SOD and CAT, as intracellular oxygen free radical scavenging enzymes, can resist the damage of superoxide anions and decompose hydrogen peroxide, reducing oxidative damage to cellular DNA [26]. The results showed that the BspC protein inhibited the activities of SOD, CAT and GSH and increased the activity of MDA, thereby inhibiting the antioxidant capacity of the host cells.

In conclusion, while BspC appears unique in its strong pro-apoptotic and pro-inflammatory phenotype among the *Brucella* effector repertoire, its functions are likely not isolated. It may act coordinately or sequentially with other effectors, such as BspB in Golgi manipulation or VceC in ER stress induction, to finely orchestrate host cell processes – first by disrupting homeostasis through stress and inflammation, and later by ensuring bacterial replication survival. This potential collaboration underscores the complexity of *Brucella*’s virulence strategy, where the combined action of effectors with seemingly opposing functions achieves a delicate balance crucial for infection.

## Abbreviations

BCV: *Brucella*-containing vacuole
BP: biological process
CAT: catalase
CC: cellular component
DEGs: differentially expressed genes
GSH: glutathione
MDA: malondialdehyde
MF: molecular function
qRT-PCR: quantitative reverse transcription PCR
SOD: superoxide dismutase
